# Supplementary material for: The Rauvolfia tetraphylla genome suggests multiple distinct biosynthetic routes for yohimbane monoterpene indole alkaloids
Source: Commun Biol. 2023 Nov 24;6:1197. doi: 10.1038/s42003-023-05574-8 (PMC10673892; doi:10.1038/s42003-023-05574-8)
Supplement: Supplementary file 2 — Supplementary Information [file 42003_2023_5574_MOESM2_ESM.pdf]

Supplementary Information for “The *Rauvolfia tetraphylla* genome suggests distinct biosynthetic routes for yohimbane monoterpene indole alkaloids”

By Emily Amor STANDER et al.

Supplementary Figures:

- Supplementary Figure S1 2
- Supplementary Figure S2 3
- Supplementary Figure S3 3
- Supplementary Figure S4 4
- Supplementary Figure S5 5
- Supplementary Figure S6 6
- Supplementary Figure S7 7
- Supplementary Figure S8 7
- Supplementary Figure S9 8
- Supplementary Figure S10 8
- Supplementary Figure S11 9

Supplementary Tables

- Supplementary Table S1 10
- Supplementary Table S2 10
- Supplementary Table S3 11
- Supplementary Table S4 11
- Supplementary Table S5 12
- Supplementary Table S6 13
- Supplementary Table S7 14

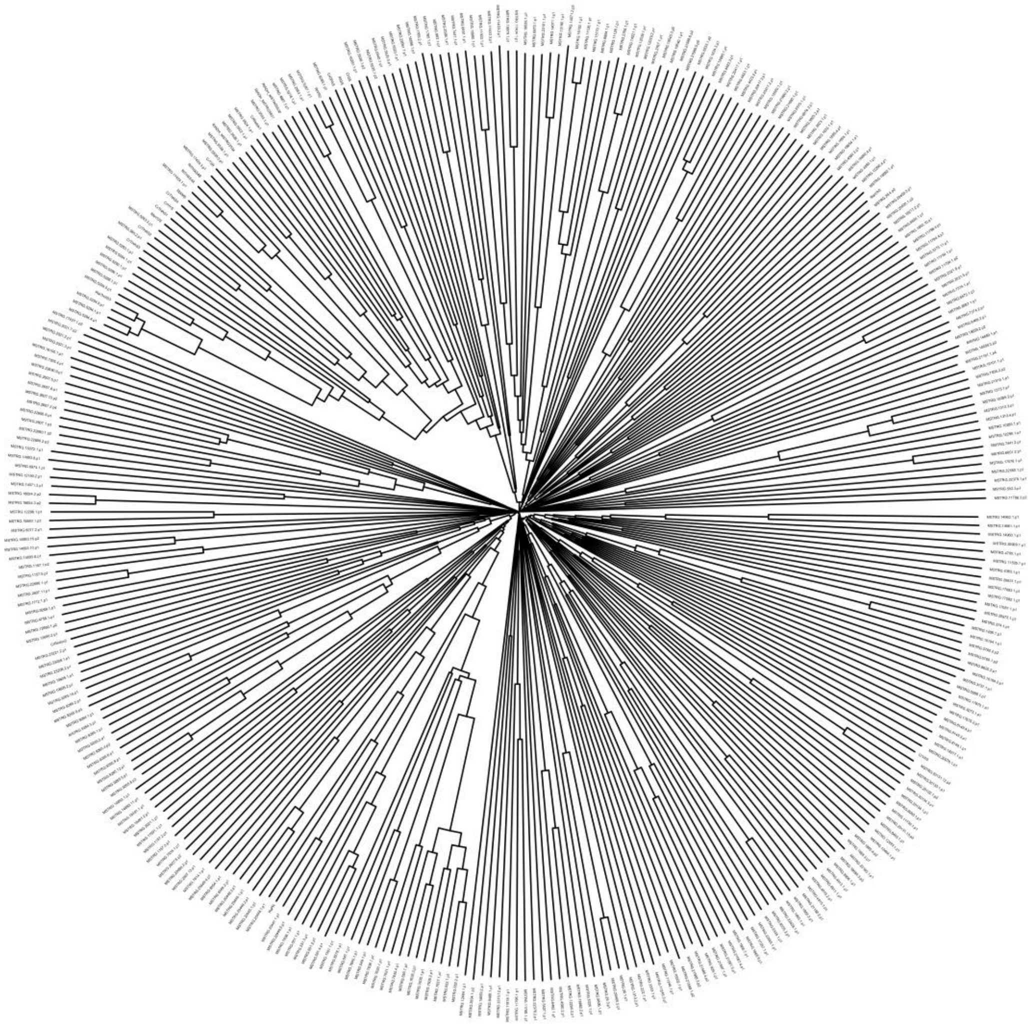

Supplementary Figure S1. Phylogenetics tree of ADH-coding proteins from R. tetraphylla and other ADHs involved in MIA biosynthesis. Proteins were aligned using muscle v.3.6. Sequence distance was calculated using dist.alignment function implemented in ape package (v.5.7.1) and an unrooted neighbor-joining tree was constructed from this distance matrix using nj function implemented in ape package (v.5.7.1) and 1,000 bootstrap were performed using boot.phylo function implemented in ape package (v.5.7.1).

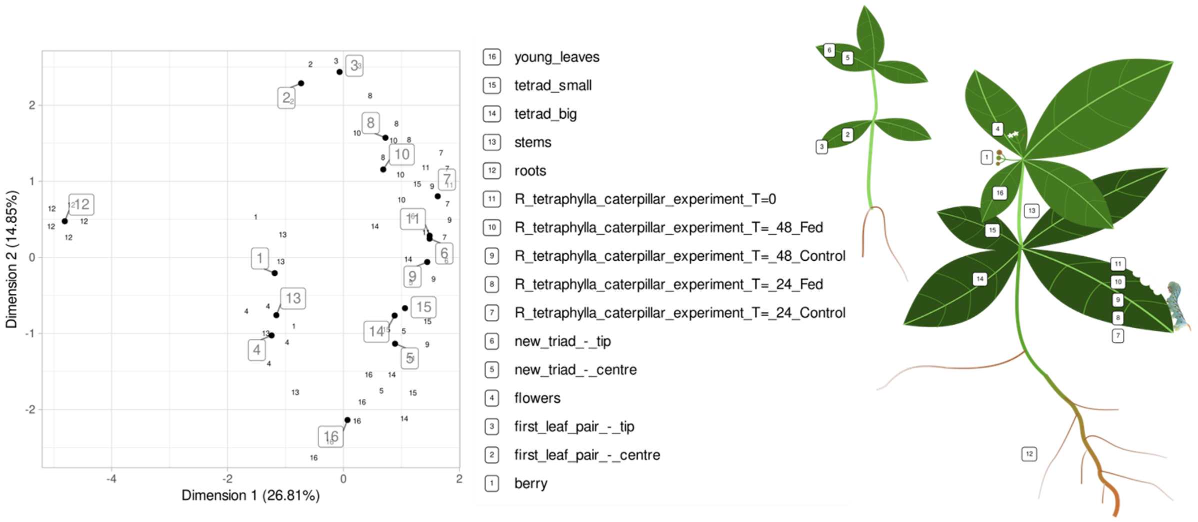

Supplementary Figure S2. Multidimensional scaling of gene expression

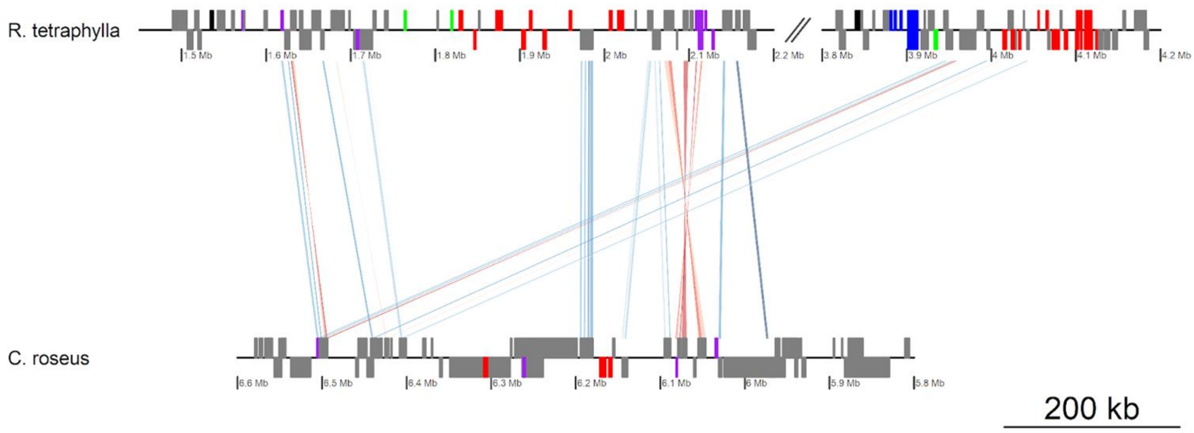

Supplementary Figure S3. **Synteny between R. tetraphylla ADH clusters and C. roseus genome v.2.1 contig 884**. Red boxes: putative alcohol dehydrogenases, Black bordered red boxes: ADH candidates, purple boxes: cytochrome P450, blue boxes: UDP-glycosyltransferases, green boxes: MIA enzyme orthologs, black boxes: transcription factors, gray boxes: other functions.

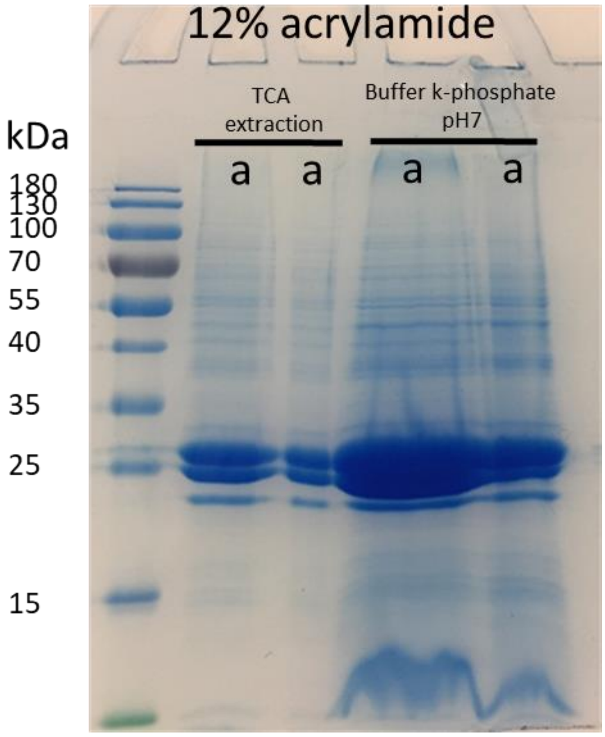

Supplementary Figure S4. SDS-PAGE gel. TCA extraction: SDS-extraction buffer (40mM Tris, 2% SDS, 60 mM DTT), after a TCA/acetone precipitation and finally ressuspension of the pelleted proteins in loading buffer 1x. Buffer k-phosphate pH7: dilution of the latex (1V: 1V) in 50mM K-phosphate buffer pH 7. Loading buffer : 40%v/v glycerol, 250 mM Tris-HCl pH 6.8, 8% SDS, 5% β-mercaptoethanol

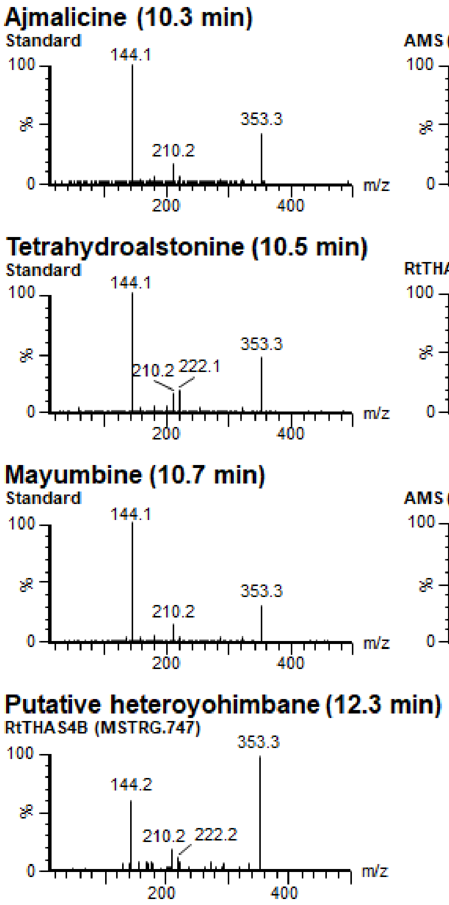

Supplementary Figure S5. MS-MS fragmentation of heteroyohimbane standards compared to m/z 353 products of *R. tetraphylla* ADHs.

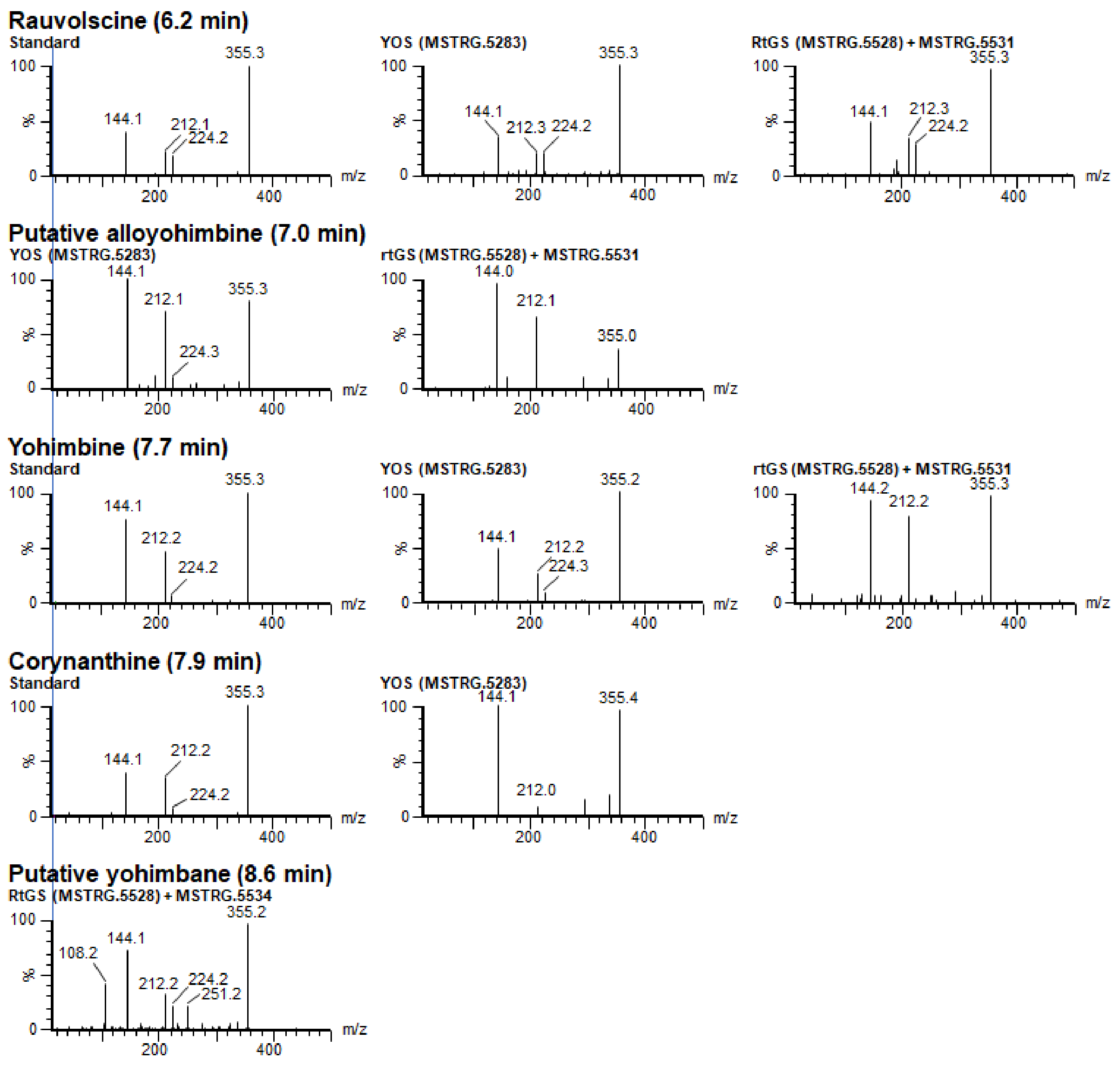

Supplementary Figure S6. MS-MS fragmentation of yohimbane standards compared to m/z 355 products of *R. tetraphylla* ADHs.

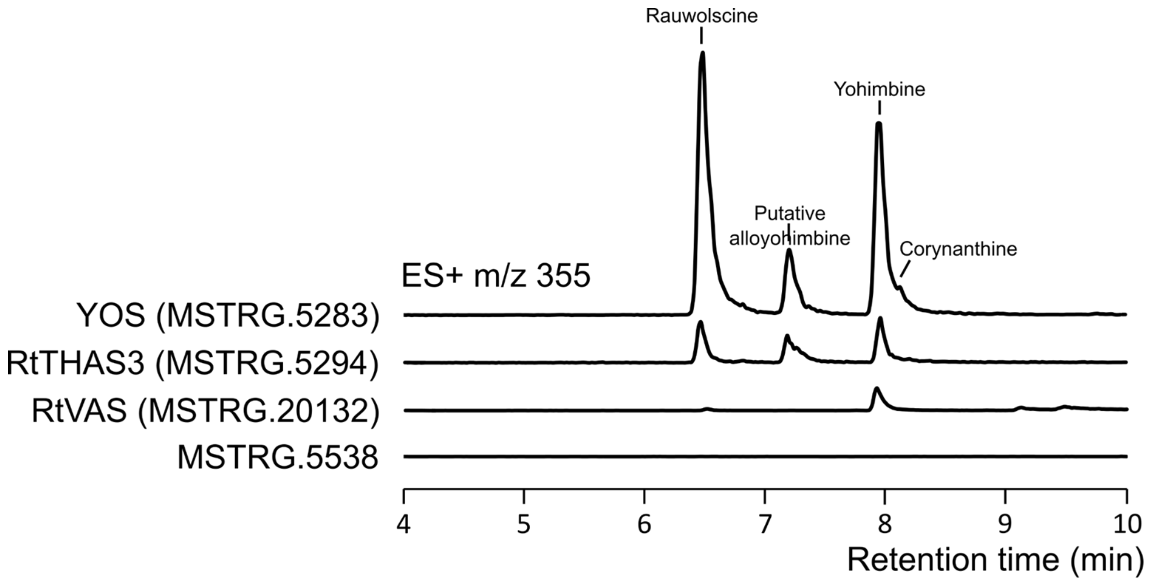

Supplementary Figure S7. Compared yohimbane production of YOS, RtTHAS3 and RtVAS as complement of Figure 10. MSTRG.5538 was used as negative control.

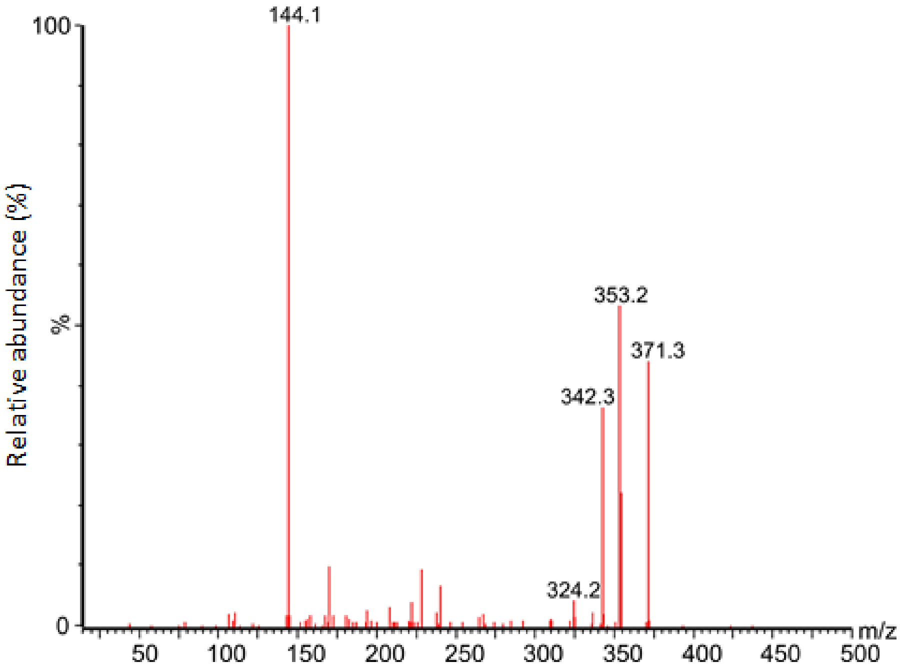

Supplementary Figure S8. MS-MS fragmentation of m/z 371 product of RtVAS (MSTRG.20132). Daughter ions 353, 342, 324 and 144 are reported for vitrosamine (Stavrinides et al., 2018).

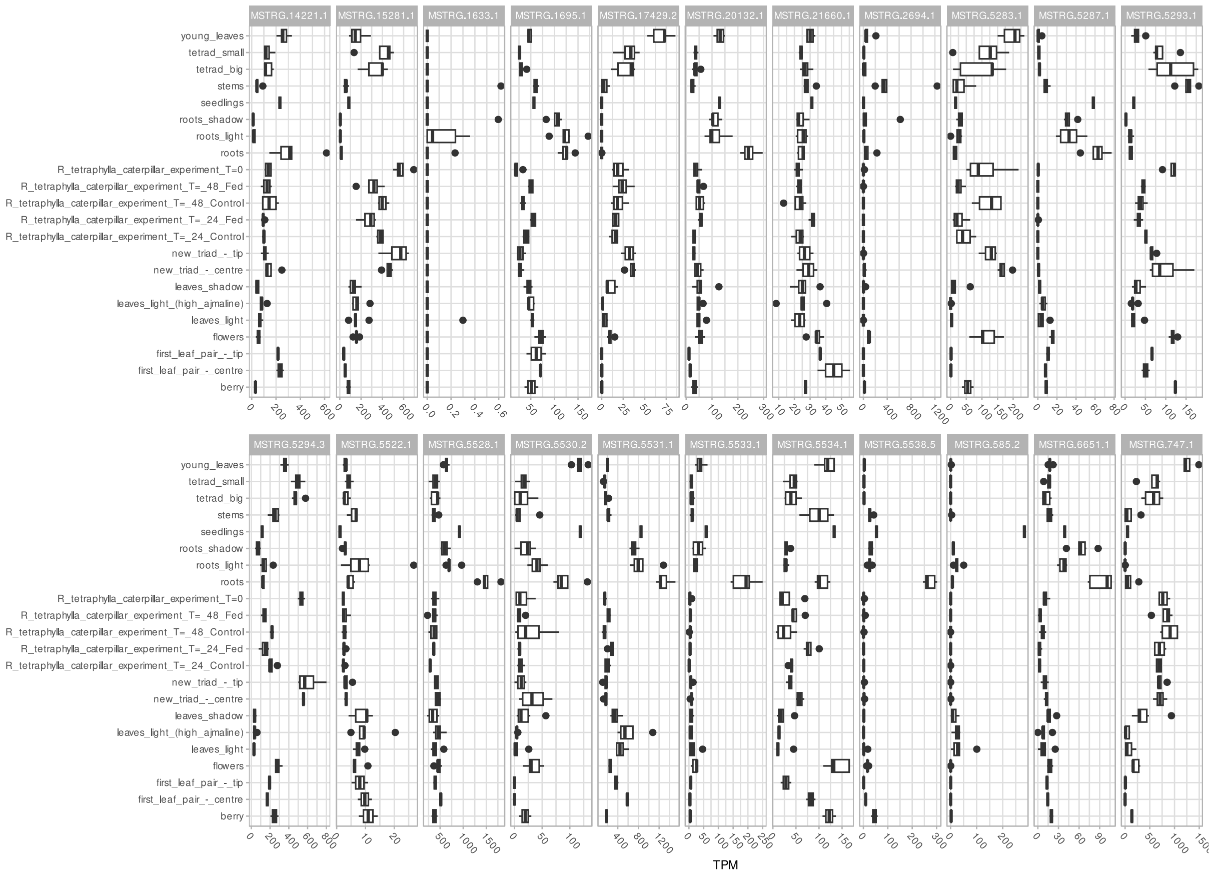

Supplementary Figure S9. Expression profiles of the different ADH candidate genes

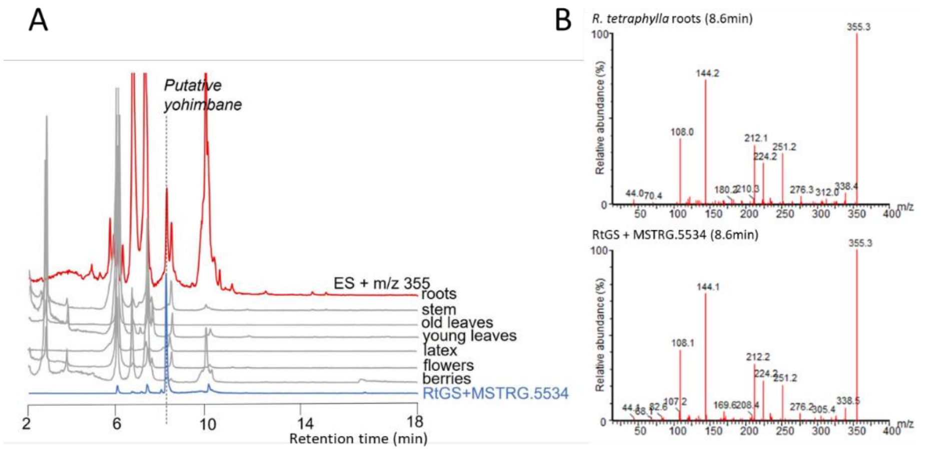

Supplementary Figure S10. Comparison of MSTRG.5534 yohimbane products with *R. tetraphylla* metabolic profiles. (A) The putative yohimbane produced by MSTRG.5538 (in positive ionisation mode at m/z 355 and retention time of 8.6 min) is only present in roots of *R. tetraphylla*. (B) MS/MS fragmentation of the compound of interest form MSTRG.5534 reaction mix and roots of *R. tetraphylla*.

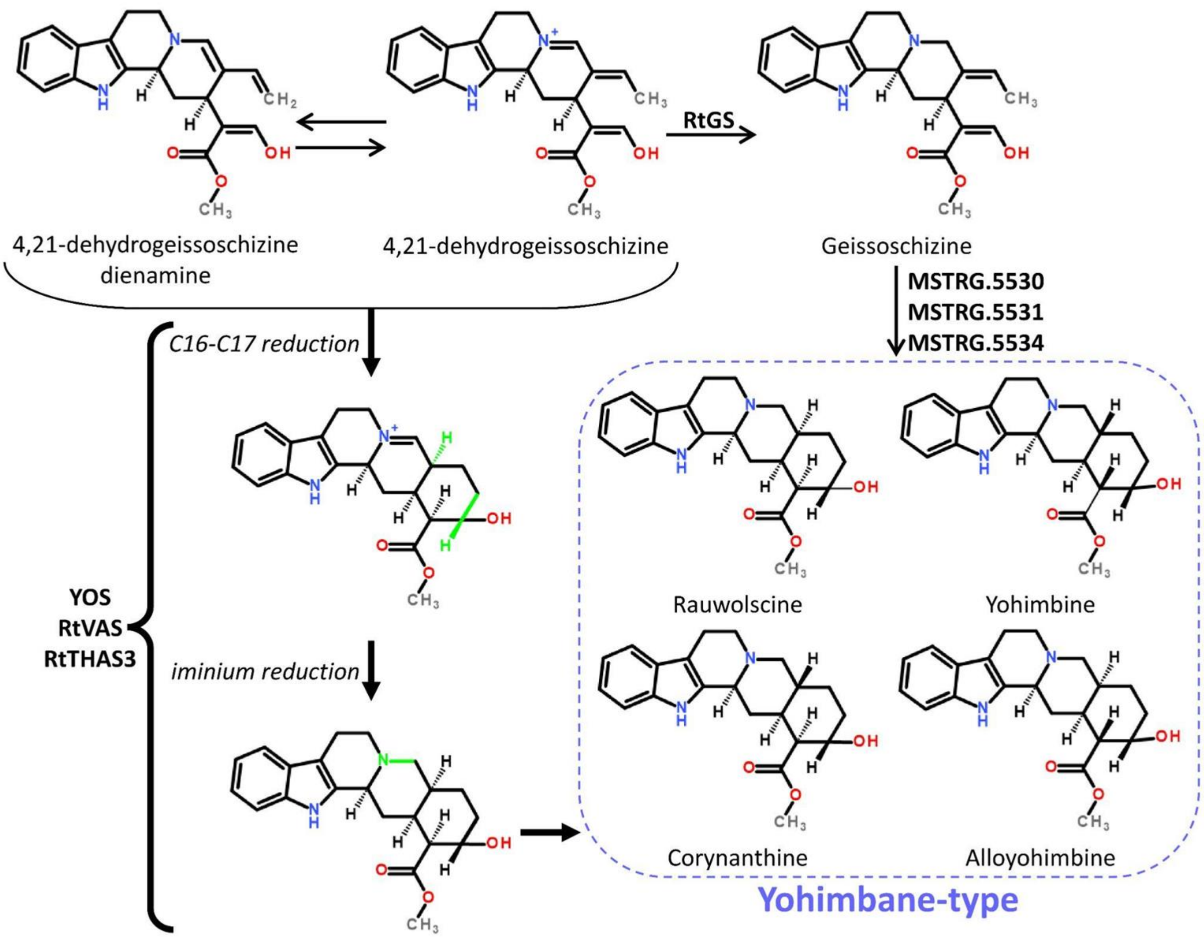

Supplementary Figure S11. Possible mechanistic scenario for rearrangement of 4, 21-dehydrogeissoschizine to yohimbanes involving one enzyme (YOS, RtVAS, RtTHAS3) or two distinct enzymes (RtGS and MSTRG.5530, MSTRG.5531 or MSTRG.5534).

Supplementary Table S1. Genome assembly features of *Rauvolfia tetraphylla*

Length of genome assembly (bp)	364,945,498
Number of scaffolds	76
N50 of scaffolds (Mb)	8.135
L50 of scaffolds	18
GC content (%)	33.89%
Longest scaffold (Mb)	15.726
Fraction of genome in >50kb scaffolds	76
QV	32.9546
LTR assembly index (raw)	18.90 (13.27)
Genome BUSCO (Complete[Single-Copy, Duplicated], Fragmented, Missing)	96.2[79.8, 16.4], 1.3, 2.5
Total length of long-terminal repeat transposable elements (bp)	124,646,167
Total length of terminal inverted repeat transposable elements (bp)	904,136
Number of protein-coding genes	23,228
Annotated gene BUSCO (Complete[Single-Copy, Duplicated], Fragmented, Missing)	93.9[72.5, 21.4]n 1.9, 4.2
Average gene length	6,06
Average transcripts number per gene	45018
Average exon length	296
Average exon number per transcript	44964

Supplementary Table S2. Number of ADH families in the different MIA-producing Apocynaceae species

ADH family	*R. tetraphylla*	*C. roseus*	*V. minor*	*V. thouarsii*
**SDR**	76	59	20	25
**MDR**	79	62	74	72
**AKR**	27	26	32	50
**Total**	182	147	126	147

Supplementary Table S3. Transposable element annotation metrics

Family	Class	Count	bpMasked	%masked
**LTR**	**Copia**	90341	73868386	20.24%
	**Gypsy**	32066	24805528	6.80%
	**unknown**	54502	25972253	7.12%
**TIR**	**CACTA**	857	172722	0.05%
	**Mutator**	944	348161	0.10%
	**PIF_Harbinger**	311	80717	0.02%
	**Tc1_Mariner**	4	1134	0.00%
	**hAT**	630	295159	0.08%
	**unknown**	31	6243	0.00%
**nonLTR**	**LINE_element**	1045	802622	0.22%
	**unknown**	183	68073	0.02%
**nonTIR**	**helitron**	55960	17020937	4.66%
**repeat_region**		55973	14460882	3.96%
	**Total**	292847	157902817	43.27%

Supplementary Table S4. Supporting data for Fig.6c.

epochs	training_logloss	validation_logloss
10	0.144758950666082	0.121029346843042
360	0.0888721097854829	0.106896004711185
730	0.0705336568964392	0.105252958984825
1130	0.0773578302875656	0.0918092748885388
1510	0.0749282173035484	0.0876652851499177

Supplementary Table S5. Distance matrix of the different ADH candidate genes to functionally characterized ADHs.

	THAS1	*THAS2*	*THAS3*	*THAS4*	*HYS*	*GS*	*T3R*	*DPAS*	*Redox1*	*Redox2*	*VAS*
MSTRG.5294	0,5349	0,4136	0,2131	0,5435	0,5386	0,6283	0,5725	0,636	0,5986	2,207	2,3257
MSTRG.17429	0,353	0,5632	0,6089	0,3587	0,3547	0,66	0,4508	0,6654	0,5976	2,1192	2,2513
MSTRG.747	0,3514	0,5683	0,6149	0,3547	0,3507	0,66	0,4598	0,6438	0,5872	2,1466	2,2513
MSRTG.1695	1,5597	1,5447	1,5224	1,5597	1,575	1,655	1,5383	1,5597	1,6519	2,4953	2,5402
MSTRG.2694	0,2933	0,5289	0,591	0,2473	0,226	0,5867	0,3899	0,6332	0,5568	2,2676	2,1617
MSTRG.5283	0,1972	0,5732	0,6029	0,322	0,299	0,6174	0,4026	0,6227	0,5468	2,3321	2,1617
MSTRG.5528	0,5504	0,6469	0,6836	0,5766	0,5816	0,0865	0,6903	0,727	0,6194	2,366	2,1715
MSTRG.5530	0,5118	0,5547	0,6158	0,5716	0,5667	0,6234	0,6623	0,4879	0,4593	2,2368	1,9892
MSTRG.5531	0,4977	0,5596	0,6278	0,5471	0,5568	0,6131	0,6459	0,5062	0,4728	2,2368	2,1433
MSTRG.5534	0,4314	0,4859	0,558	0,4605	0,4516	0,5603	0,478	0,5127	0,4387	2,178	2,1433
MSTRG.20132	2,0561	2,1551	2,3026	2,2347	2,2039	2,2139	2,2039	2,3431	2,0995	2,3132	0,1569

Supplementary Table S6. Primers used in this study

	**Forward**	**Reverse**
MSTRG.747	ctgagaggatccATGGCTCCGAATTCGCCTGA	ctgagaggatccTTATGCAGATTTGAGTGTGTTTCC
MSTRG.1695	ctgagaggatccATGATGGTCAAGGCTATCAG	ctgagaggatccCTACTGATCAATGGTTGG
MSTRG.2694	ctgagaggatccATGGCTGATGAAATGTCTG	ctgagaggatccTCATGCAGATTTGAGGCAG
MSTRG.5283	ctgagaggatccATGAGGTTATGTTGTCAGTATAAGAAGTCCTC	ctgagaggatccTTATGCAGATCCGAGAGTGTTTTCCATG
MSTRG.5294	ctgagaggatccATGGCAGCAGCAGAAACAGCA	ctgagaggatccCTAAGCAGATTTCAATGTATTGCCTATATCAACC
MSTRG.5528	ctgagaggatccATGGCTGGGGAAACAACCCAA	ctgagaggatccTCAGTCCTCATATTTCAACGTATTTCCA
MSTRG.5530	ctgagaggatccATGGCCAGGAAATCACCAGAAGAG	ctgagaggatccTTAGGGAGCTCTCAAGGTGTTGGC
MSTRG.5531	ctgagaggatccATGGCCGCAAAATCACCGGAA	ctgagaggatccTTAAGGAGCTTTCAAGGTGTTGGCTACGTC
MSTRG.5534	ctgagaggatccATGGCGAAGTCACCAGAAGTTGAG	ctgagaggatccTTAGGCAGATTTCAATGTGTTGCC
MSTRG.5538	ctgagaggatccATGGCGATCTCACCAGAAGTTGAG	ctgagaggatccTTAAGCAGATTTCAATGTGTTGCAAATG
MSTRG.17429	ctgagaggatccATGTGCAGCTCAGGGAG	ctgagaggatccTTATGCAGATTTGAGTGTG
MSTRG.20132	ctgagaagatctATGGCGGCGGTGGCAGCACAACATT	ctgagaagatctTCACTCATACGATGACTCCTCACTGC
**BJL166 Universal Backbone F**	AAGGAAGGAGUTAGACAACCTGAAGTCTAGG	AGTAGATAAUTACTTCCTTGATGATCTG
**BJL167 Universal Gene**	ATTATCTACUTTTTACAACAAATATAAAACAATCTG	ACTCCTTCCTUTTCGGTTAGAGCGGATGAATGCACG

Supplementary Table S7. Plasmid constructs used in this study

**pBJL118**	pTEF1-RseSDG-tADH + pPGK1-MSTRG.5283-tCYC1
**pBJL119**	pTEF1-RseSDG-tADH + pPGK1-MSTRG.5528-tCYC1
**pBJL120**	pTEF1-RseSDG-tADH + pPGK1-MSTRG.585-tCYC1
**pBJL121**	pTEF1-RseSDG-tADH + pPGK1-MSTRG.5538-tCYC1
**pBJL122**	pTEF1-RseSDG-tADH + pPGK1-MSTRG.5293-tCYC1
**pBJL123**	pTEF1-RseSDG-tADH + pPGK1-MSTRG.5530-tCYC1
**pBJL124**	pTEF1-RseSDG-tADH + pPGK1-MSTRG.5531-tCYC1
**pBJL125**	pTEF1-RseSDG-tADH + pPGK1-MSTRG.5533-tCYC1
**pBJL126**	pTEF1-RseSDG-tADH + pPGK1-MSTRG.5534-tCYC1
**pBJL127**	pTEF1-RseSDG-tADH + pPGK1-MSTRG.5287-tCYC1
**pBJL128**	pTEF1-RseSDG-tADH + pPGK1-MSTRG.14221-tCYC1
**pBJL129**	pTEF1-RseSDG-tADH + pPGK1-MSTRG.2694-tCYC1
**pBJL66**	pTEF1-RseSDG-tADH + pPGK1-MSTRG.17429-tCYC1
**pBJL67**	pTEF1-RseSDG-tADH + pPGK1-MSTRG.747-tCYC1
**pBJL68**	pTEF1-RseSDG-tADH + pPGK1-MSTRG.20132-tCYC1
**pBJL69**	pTEF1-RseSDG-tADH + pPGK1-MSTRG.1633-tCYC1
**pBJL70**	pTEF1-RseSDG-tADH + pPGK1-MSTRG.1695-tCYC1
**pBJL74**	pTEF1-RseSDG-tADH + pPGK1-MSTRG.15281-tCYC1
**pBJL75**	pTEF1-RseSDG-tADH + pPGK1-MSTRG.5294-tCYC1
**pBJL133**	pTEF1-RseSDG-tADH + pPGK1-MSTRG.6651-tCYC1
**pBJL138**	pTEF1-RseSDG-tADH + pPGK1-MSTRG.21660-tCYC1
**pBJL143**	pTEF1-RseSDG-tADH + pPGK1-MSTRG.5522-tCYC1
