# Supplementary material for: The Rauvolfia tetraphylla genome suggests multiple distinct biosynthetic routes for yohimbane monoterpene indole alkaloids
Source: Commun Biol. 2023 Nov 24;6:1197. doi: 10.1038/s42003-023-05574-8 (PMC10673892; doi:10.1038/s42003-023-05574-8)
Supplement: Supplementary file 3 — Description of Addiitonal Supplementary Files [file 42003_2023_5574_MOESM3_ESM.pdf]

Description of Additional Supplementary Files

File name: Supplementary Data S1.

Description: Gene functional annotation

File name: Supplementary Data S2.

Description: MIA biosynthetic genes orthologs

File name: Supplementary Data S3.

Description: Synonymous substitution rate for gene paralogs in A. thaliana, C. roseus, O. pumila, R. tetraphylla, S. lycorpersicum and V. minor.

File name: Supplementary Data S4.

Description: Enriched GO terms in R. tetraphylla expanded orthogroups

File name: Supplementary Data S5.

Description: Sample description and RNAseq reads metrics

File name: Supplementary Data S6.

Description: MIA quantification in the different R. tetraphylla organs

File name: Supplementary Data S7.

Description: Candidate genes list from co-expression analysis

File name: Supplementary Data S8.

Description: Candidate genes list from deep learning analysis

File name: Supplementary Data S9.

Description: Candidate genes list from latex proteomics analysis

File name: Supplementary Data S10.

Description: MIA biosynthetic gene clusters

File name: Supplementary Data S11.

Description: ADHs in MIA biosynthetic gene clusters

File name: Supplementary Data S12.

Description: Summary of ADH candidate genes

File name: Supplementary Data S13.

Description: Supporting data for figure 9

File name: Supplementary Data S14.

Description: Supporting data for figure 10
